# Transition from laparoscopic to robotic hepatectomy within a minimally invasive liver surgery program: risk-adjusted learning curve assessment by multiple continuous outcomes

**DOI:** 10.1007/s11701-026-04004-1

**Published:** 2026-09-27

**Authors:** Lorenzo Bernardi, Eleonora Garosi, Raffaello Roesel, Francesco Mongelli, Isabella Hunjan, Virginia Sitta, Pietro Majno-Hurst, Alessandra Cristaudi

**Affiliations:** 1 Department of Surgery, Lugano Regional Hospital, Ente Ospedaliero Cantonale (EOC), Via Tesserete 46, 6900 Lugano, Switzerland; 2 Faculty of Biomedical Sciences, University of Southern Switzerland (USI), Lugano, Switzerland

**Keywords:** Minimally invasive liver surgery, Hepatectomy, Robotic surgery, Risk-adjusted learning curve.

## Abstract

Training in minimally invasive liver surgery (MILS) usually encompasses prior laparoscopic liver resection (LLR) experience before moving to robotic liver resections (RLR). We analyzed the risk-adjusted learning curve of MILS within the transition from LLR to RLR. Consecutive LLR and RLR from a surgeon trained sequentially in the two techniques were reviewed. Multiple regression analysis was used to adjust for patient- and hepatectomy-related confounders. The cumulative sum (CUSUM) analysis of the risk-adjusted operative time, blood loss, and comprehensive complication index (CCI) was used to estimate the learning curves based on competency, proficiency and mastery phases. One-hundred-ninety-seven consecutive MILS were reviewed (LLR = 97; RLR = 100). The first RLR was case 74, after which the use of LLR decreased sharply. A steady improvement of adjusted operative time and blood loss continued while introducing RLR. For the whole body of MILS, competency was obtained after 90 and 111 cases based on the adjusted operative time and blood loss respectively, proficiency followed after a similar number of cases. The separate analysis of LLR and RLR showed earlier phase transition for RLR: adjusted operative time and blood loss dropped after 38 and 45 LLRs, while they improved earlier after 19 and 29 RLRs. Risk-adjusted CCI improved later in the mastery phase in both groups. A shorter learning curve was observed for RLR following prior LLR experience. Risk-adjusted improvements occurred earlier in operative time (after 38 LLR vs. 19 RLR) and blood loss (45 LLR vs. 29 RLR) than for postoperative morbidity. Careful stepwise introduction of RLR did not deteriorate the surgical performance while transitioning from LLR to RLR.

**Supplementary Information:**

The online version contains supplementary material available at https://doi.org/10.1007/s11701-026-04004-1.

## Introduction

Minimally invasive liver surgery (MILS) gradually became the standard approach in selected patients with resectable primary and secondary liver tumors [1]. While maintaining open liver resection (OLR) as the reference, many programs gained experience in laparoscopic liver resections (LLR) and more recently in robotic liver resections (RLR) [2].

Robotic liver surgery overcame the limitations of laparoscopy and showed benefits in short-term outcomes (shorter operative time, less risk of bleeding and conversion to laparotomy), without compromising the oncologic results compared to both LLR and OLR [3–5]. Moreover, the robotic approach was instrumental in expanding the use of MILS to a larger pool of patients otherwise rarely approached with laparoscopy, like those with tumors in the posterior-superior segments, cirrhotic patients with portal hypertension, those needing repeat hepatectomy or biliary-enteric reconstruction [6–9].

Both LLR and RLR remain challenging procedures and understanding the process needed to acquire competency is key for their safe and genuine diffusion [10]. Based on the IDEAL framework (Idea, Development, Exploration, Assessment and Long-term Monitoring) proposed to guide the safe diffusion of surgical innovations, both LLR and RLR stand in the exploration and assessment stages [11, 12]. As such, studies are expected to focus on the intervention context and learning curves by using standardized metrics and accounting for specific confounders [13].

The learning curve of MILS has been studied under various conditions and settings, but the long-standing debate over the most rigorous methodology for its assessment continues [14, 15]. Adding further complexity, the surgical backgrounds and the training of hepatobiliary surgeons embracing RLR have changed in recent years: the pioneers of laparoscopic hepatectomy started RLR after many years of LLR experience, whereas younger surgeons are moving to RLR faster, being trained directly in RLR or sometimes in the two techniques concomitantly [16–19].

Studies on the learning curve of MILS should employ robust statistical methods and account for potential sources of bias related to patient- or hepatectomy-related factors for reliable and reproducible measures (risk-adjusted learning curve) [15, 20]. Besides the above-mentioned points, defending patients’ safety and maintaining favorable clinical outcomes is non-negotiable when introducing a new surgical technique [21].

The objective of the study was to analyze the risk-adjusted learning curve of MILS during the sequential training in LLR and then RLR, a common scenario in liver surgery to date.

## Methods

### Study design and setting

This was an observational retrospective cohort study within the Department of Surgery at Lugano Regional Hospital, a tertiary center for hepatobiliary and pancreatic surgery in Switzerland. The study was compliant with the STROCSS guidelines for reporting cohort studies in surgery [22]. A single operating surgeon (A.C.), already proficient in general surgery and OLR, was trained sequentially in LLR then in RLR. The risk-adjusted surgical performance over time was analyzed. To capture the effect of introducing robotic surgery in the program, the risk-adjusted learning curve was analyzed in consecutive MILS and in LLR and RLR separately. A recent proposal for standardized 3-phases (competency, proficiency, mastery) reporting of learning curves was endorsed [15].

### Participants

Consecutive elective LLR and RLR performed by a single surgeon from May 2018 to December 2025 were included. Non-elective resections, OLR, MILS performed by other surgeons and cysts fenestrations were excluded.

### Ethical approval and informed consent statement

The study was approved by the regional ethical committee (Comitato Etico Cantonale del Ticino, Lugano, Switzerland, project-ID 2025 − 00195). All patients were provided with an informed general consent or non-objection letter for participating in the study.

### Selection of MILS candidates

The background in general and liver surgery of the operating surgeon has been described in a prior pilot study, at that time we were performing LLR - but not RLR - in our center [19]. Briefly, training in laparoscopic hepatectomy was initiated under the mentoring of a senior hepatobiliary surgeon from our institution. The training in robotic surgery started in early 2023 with general surgery cases including inguinal hernia repair and cholecystectomy and was preceded by participation in internationally accredited courses for RLR.

Both LLR and RLR started with limited resections of superficial tumors before approaching major hepatectomies, tumors close to vascular or biliary structures and repeat hepatectomies. The transition towards RLR depended solely on the availability of the robotic platform (1–2 times per week, shared with other general surgery specialties). LLR (freely available) was not completely abandoned and was used only when the robotic platform was not available. OLR remained the approach of choice for cases requiring biliary/vascular reconstructions or very large tumors (i.e., > 10 cm).

### Surgical technique

#### LLR:

four or five laparoscopic trocars were placed along a J-shaped line depending on the type of resection planned. Parenchymal transection was performed with the laparoscopic Cavitron Ultrasonic Surgical Aspirator (CUSA^©^) [4].

#### RLR:

the Da Vinci Xi^©^ robotic system was used. Four robotic trocars (8 mm) were placed along a horizontal line, with one assistant laparoscopic trocar. Parenchymal transection was performed with the clamp-crush technique using the robotic bipolar forceps or the Maryland^©^ bipolar forceps and a sealing device (Synchro Seal^©^). Specimens were removed via small suprapubic or midline incisions in both LLR and RLR. The principles of parenchymal sparing surgery (PSS) were applied whenever appropriate [23].

### Outcomes

The primary outcome of the study was the risk-adjusted learning curve, defined as the number of operations required to achieve an improvement in the surgical performance while adjusting for patient characteristics and hepatectomy complexity. The surgeon’s performance was surrogated by 3 target continuous variables: *(a)* adjusted operative time, *(b)* adjusted blood loss, and *(c)* adjusted comprehensive complication index (CCI) [24]. The raw intra- and postoperative outcomes of LLR and RLR were the secondary outcomes.

### Data source and measurements

Data were extracted from the prospectively collected institutional database and included patients’ characteristics, surgical procedures and outcomes. Intraoperative data included the type of hepatectomy (Brisbane nomenclature), use of lymphadenectomy, resection of neighbor organs, operative time (skin incision to skin closure), blood loss, and conversions to laparotomy [25]. The Iwate score was used to grade the complexity of MILS and was based on the most complex resection in case of multiple resections [26]. Postoperative data included morbidity and major morbidity within 90 days after surgery based on the Clavien-Dindo and CCI gradings [24, 27]. Biliary leak and post-hepatectomy liver failure were defined in accordance with The International Study Group for Liver Surgery (ISGLS) [28, 29]. Textbook outcomes were defined according to Gorgec et al. [30].

### Statistical analysis

Descriptive statistics were presented as frequencies for categorical variables. Continuous variables were reported as mean with standard deviation (SD) or median with interquartile ranges (IQR) based on their distribution. Comparisons between LLR and RLR were performed with the Fisher or Chi-square tests for dichotomous variables and the Mann-Whitney test for continuous variables. For the main binary postoperative outcomes unadjusted risk ratios (RR) with 95% confidence intervals were reported. The statistical significance level was set at 5% (*p* < 0.05).

A risk-adjusted learning curve model was chosen to minimize the risk of bias. A multiple regression analysis was used to identify relevant confounders and to adjust the target outcomes in the whole body of MILS and in LLR and RLR separately. The same covariates were included in the regression models: age, sex, BMI, Charlson comorbidity index, cirrhosis, previous liver surgery, number of resection areas, Iwate score, extended resection to neighbor organs, concomitant ablation and locoregional lymphadenectomy. Models fit was tested using R², adjusted R² and residual diagnostics (available from the Supplementary Materials, Table S1). The cumulative sum (CUSUM) method was used to estimate the learning curve by analyzing regression residuals of operative time, blood loss and CCI ordered chronologically. Operative time and blood loss were log10-transformed, while CCI was transformed as log10(CCI + 1) to retain zero values.

The two-sided CUSUM plot consists of a positive (upper) curve and a negative (lower) one. The upper curve represents the cumulative sum of positive differences between observed values and the reference; the lower one represents the cumulative sum of negative differences. Zero was used as the reference value and control limits were defined according to the variability of the corresponding residuals. Each curve indicates when the process is yielding results above or below the reference value. The combination of these curves visually depicts the performance of the surgeon over time. Phase transitions were primarily identified by visual assessment of changes in the CUSUM trajectory followed by sustained stabilization of performance. A segmented regression was used as a complementary sensitivity analysis around each transition to assess the robustness of the visually identified phase boundaries.

All analyses were conducted with MedCalc^®^ Statistical Software version 20.115 (MedCalc Software Ltd, Ostend, Belgium; https://www.medcalc.org; 2022) and SPSS statistics, version 29.0.2.0.

## Results

Two-hundred-eighty-eight patients underwent partial hepatectomy (OLR = 76; MILS = 212). Of those, patients operated by other surgeons (*n* = 32) and OLR performed by the surgeon whose learning curve was investigated (*n* = 59) were excluded. Finally, 197 patients were included in the analysis (LLR = 97; RLR = 100) (Supplementary materials, Figure S1).

### Descriptive data

Detailed patients’ characteristics were reported in Table 1. Median (IQR) age and Charlson comorbidity index were 68 (59–76) vs. 67 (58–73) (*p* = 0.294) and 7 [5–9] vs. 7 [6–9] (*p* = 0.663) in LLR vs. RLR respectively. Cirrhosis was present in 24 (24.7%) and 27 (27.0%) (*p* = 0.605) and was associated with portal hypertension in 5 (5.2%) vs. 8 (8.0%) (*p* = 0.422) of LLR vs. RLR respectively. Tumors were in posterior-superior segments in 44 (45.4%) LLR vs. 54 (54.0%) RLR cases (*p* = 0.225) while prior abdominal and liver surgery rates were 73.2% vs. 66.0% (*p* = 0.274) and 13.4% vs. 18.0% (*p* = 0.377) for LLR vs. RLR respectively. The only significant difference was observed in ASA scores, with the RLR group including more ASA III patients (50.5% in LLR vs. 69.0% in RLR; *p* = 0.016).

**Table 1:** Demographic and clinical characteristics in LLR and RLR. Dichotomous variables are expressed as number with percentage. Continuous variables are expressed as median with interquartile ranges (IQR)

Baseline data	LLR (*n* = 97)	RLR (*n* = 100)	*p*-value
Age (years), median (IQR)	68 (59–76)	67 (58–73)	0.294
Sex (male), n (%)	53 (54.6)	60 (60.0)	0.100
BMI (Kg/m^2^), median (IQR)	25 (23–27)	26 (23–28)	0.102
ASA score, n (%)			
• I	5 (5.2)	0 (0)	**0.016**
• II	40 (41.2)	29 (29.0)	
• III	49 (50.5)	69 (69.0)	
• IV	3 (3.1)	2 (2.0)	
Previous abdominal surgery, n (%)	71 (73.2)	66 (66.0)	0.274
Previous liver surgery, n (%)	13 (13.4)	18 (18.0)	0.377
Previous local treatment, n (%)	21 (21.6)	22 (22.0)	0.962
Comorbidities, n (%)			
• Cardiac disease	30 (30.9)	28 (28.0)	0.653
• Diabetes	20 (20.6)	24 (24.0)	0.650
• Chronic kidney failure	8 (8.2)	9 (9.0)	0.851
• Stroke	6 (6.2)	5 (5.0)	0.718
• Asthma	2 (2.1)	0 (0)	0.150
• Chronic obstructive pulmonary disease	7 (7.2)	11 (11.0)	0.358
Charlson Comorbidity index, median (IQR)	7 (5–9)	7 (6–9)	0.663
Cirrhosis, n (%)	24 (24.7)	27 (27.0)	0.605
• Child-Pugh A	24 (24.7)	25 (25.0)	0.372
• Child-Pugh B	0 (0)	2 (2.0)	
Portal hypertension, n (%)	5 (5.2)	8 (8.0)	0.422
Number of nodules, median (IQR)	1 (1–1)	1 (1–2)	0.225
Nodules in posterior-superior segments, n (%)	44 (45.4)	54 (54.0)	0.225

### Risk-adjusted CUSUM analysis of consecutive MILS

**Fig. 1:**
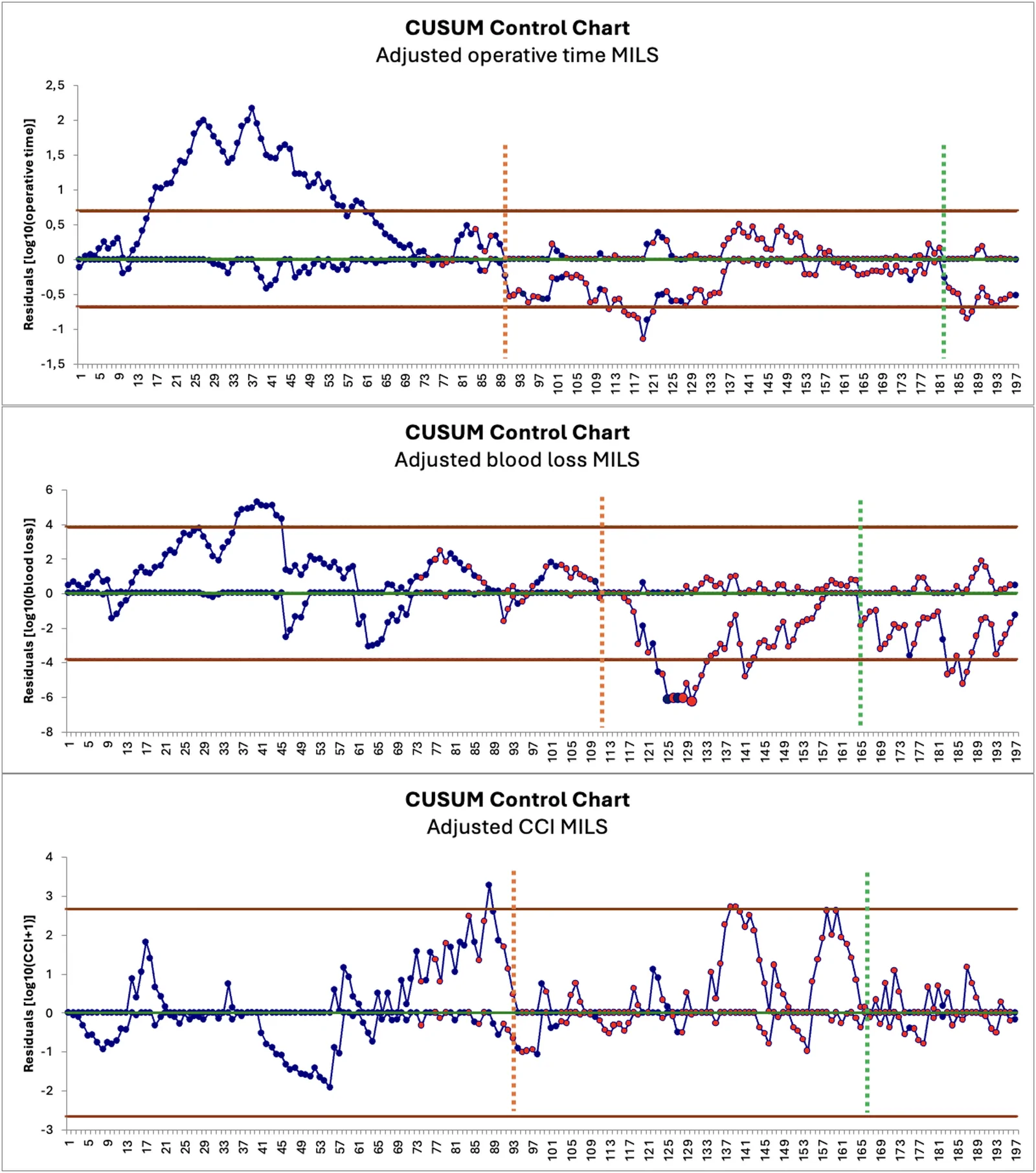
Risk-adjusted learning curve of MILS (LLR, RLR) in 197 consecutive cases, based on the CUSUM analysis of the (**a**) adjusted operative times, (**b**) blood loss and (**c**) CCI. x axis, cases; y axis, residuals; blue dots, LLR; red dots, RLR; orange dotted vertical line, transition between competency phase and proficiency phase; green dotted vertical line, transition between proficiency phase and mastery phase

The first RLR was case 74. Following the introduction of RLR, both approaches grossly co-existed until case 127; RLR drastically increased and LLR was performed sporadically thereafter (Fig. 1).

Based on the CUSUM analysis of the adjusted operative time, a steady improvement was observed after 45 LLRs and continued following the introduction of RLR, indicating that while starting a new technique (RLR) we did not observe a deterioration of the surgical performance. Three learning phases were identified as follows: competency (cases 0–90), proficiency (cases 91–182), mastery (from case 183 onwards).

A similar trend was observed for the adjusted blood loss: a steady improvement started after 45 LLRs and continued while introducing RLR. Three phases could be again identified: competency (cases 0-111), proficiency (cases 112–165), followed by the mastery phase.

In the CUSUM plot of the adjusted CCI, postoperative complications approached the reference value at the end of the competency phase (cases 0–93), then stabilized more clearly by the end of the proficiency phase (cases 94–165) (Fig. 1).

Overall, the adjusted operative time and blood loss improved sharply across the competency and proficiency phases, while the improvement in the adjusted CCI was less obvious and occurred later.

### Risk-adjusted CUSUM analysis of LLR

**Fig. 2:**
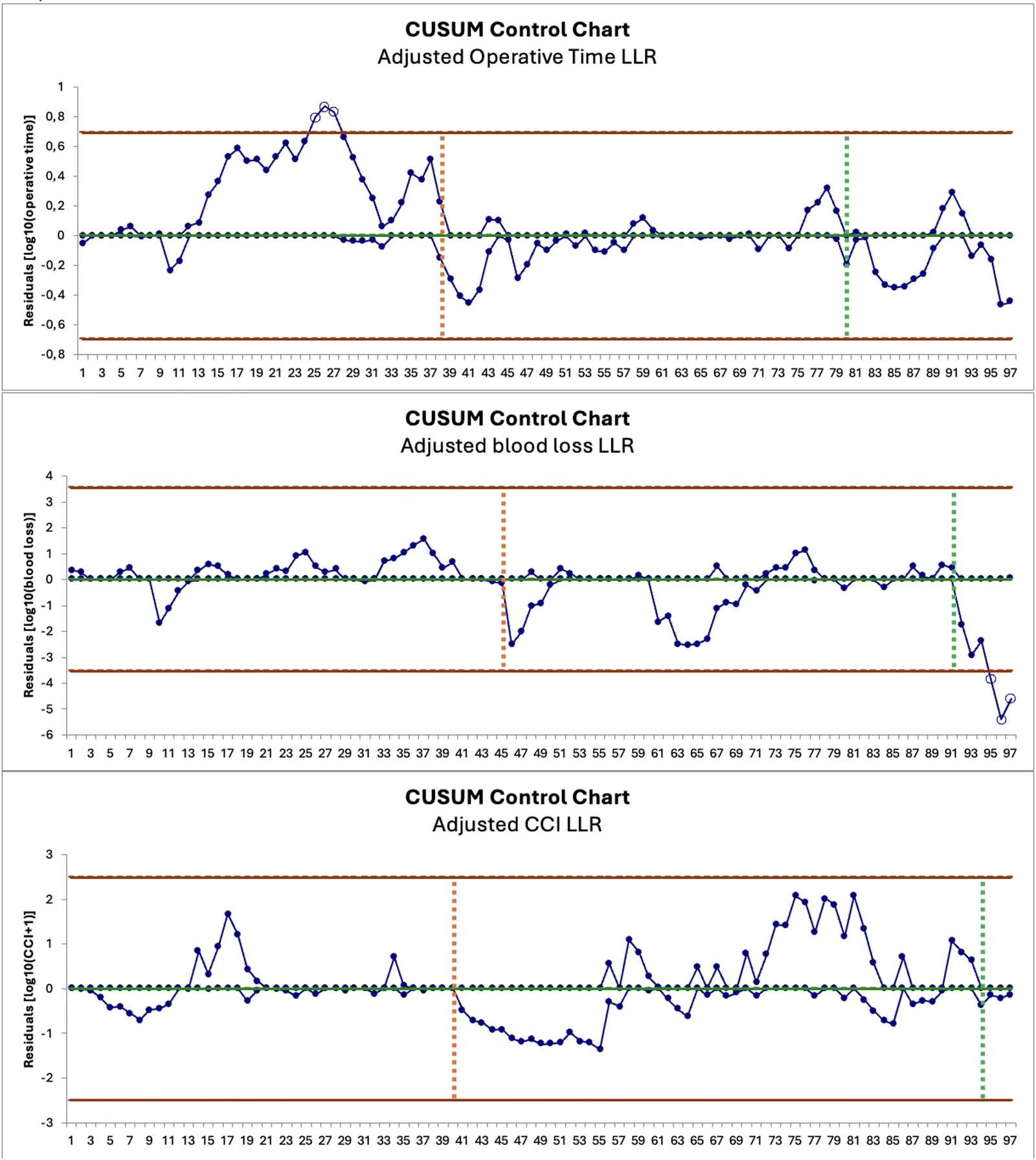
Risk-adjusted learning curve of LLR in 97 consecutive cases, based on the CUSUM analysis of the (**a**) adjusted operative times, (**b**) blood loss and (**c**) CCI. x axis, cases; y axis, residuals; blue dots, LLR; orange dotted vertical line, transition between competency phase and proficiency phase; green dotted vertical line, transition between proficiency phase and mastery phase

Based on the adjusted operative time of LLR we observed a competency phase (cases 0–38), a proficiency phase (cases 39–80), and a mastery one (from case 81 onwards). The three learning phases for the adjusted blood loss were: competency (cases 0–45), proficiency (cases 46–91), mastery (from case 92 onwards). For the adjusted CCI: competency (cases 0–40), proficiency (cases 41–94) and mastery (from case 95 onwards). (Fig. 2). The adjusted operative time and blood loss improved clearly across the competency and proficiency phases of LLR training. The improvement of the adjusted CCI occurred later and was less evident and stable.

### Risk-adjusted CUSUM analysis of RLR

Based on the adjusted operative time of RLR we observed a competency phase (cases 0–19), followed by a proficiency phase (cases 20–63), and a mastery phase (from case 64 onwards).

**Fig. 3:**
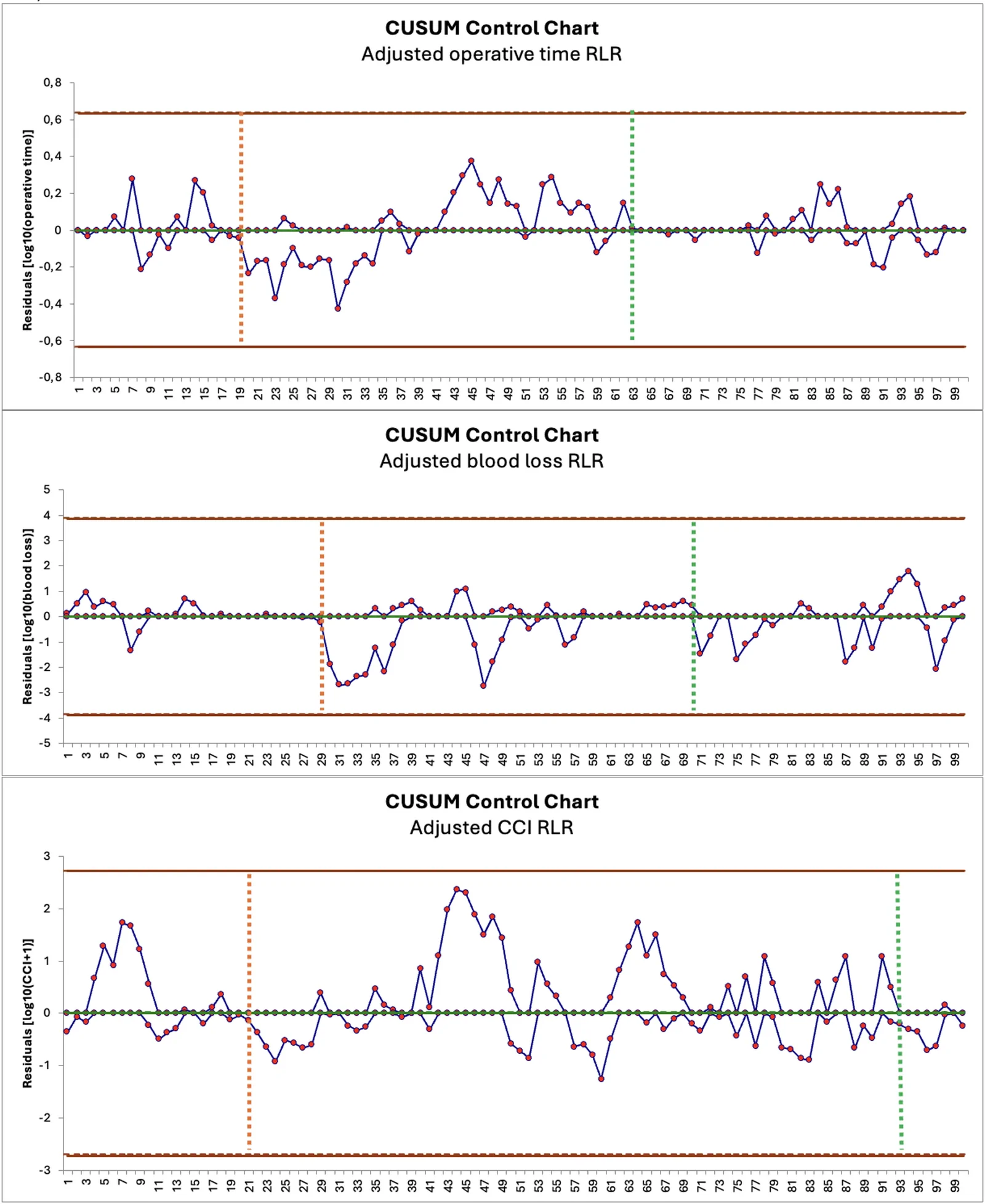
Risk-adjusted learning curve of RLR in 100 consecutive cases, based on the CUSUM analysis of the (**a**) adjusted operative times, (**b**) blood loss and (**c**) CCI. x axis, cases; y axis, residuals; red dots, RLR; orange dotted vertical line, transition between competency phase and proficiency phase; green dotted vertical line, transition between proficiency phase and mastery phase

The adjusted blood loss of RLR also dropped early in the competency phase (cases 0–29), then remained relatively stable across the proficiency phase (cases 30–70) and the mastery one (from case 71 onwards). The adjusted CCI dropped only later in the mastery phase (from case 93 onwards) (Fig. 3). In RLR, like in LLR, the adjusted operative time and blood loss improved earlier and more evidently than postoperative complications. However, earlier transition points were observed for RLR than for LLR, consistent with a shorter observed learning curve for RLR following previous LLR experience.

### Sensitivity analysis (local segmented regression)

Local segmented-regression sensitivity analyses yielded transition estimates within three procedures of the visually identified boundaries across all CUSUM curves, supporting the robustness of the 3 phases visually identified. The results of the sensitivity analysis were reported in the Supplementary Materials (Table S2).

### Transition from LLR to RLR and raw clinical outcomes

At the beginning of the study period MILS (LLR only) represented 20% of all hepatectomies. The use of LLR progressively increased to 73% before introducing RLR. Within 3 years, RLR moved from 37% up to 88% of all liver resections. In parallel, LLR and OLR decreased to 6% each. By the end of the study period MILS represented 94% of resections at our center (RLR = 88%; LLR = 6%). The role of RLR within the expansion of MILS in our program was shown in the Supplementary Materials, Figure S2.

In the crude comparison of LLR with RLR, the type of hepatectomy (*p* = 0.636) and the Iwate scores (*p* = 0.702) remained similar. Procedures consisting in multiple resections (> 1 areas of the liver) increased substantially (9.3% in LLR vs. 32.0% in RLR; *p* < 0.001); accordingly, the mean number of tumors resected was also higher in RLR (1.3 in LLR vs. 2.1 in RLR; *p* < 0.001). Compared to LLR, in RLR the use of Pringle’s maneuver (86.6% LLR vs. 32.0% RLR; *p* < 0.001) and its duration (60 min LLR vs. 35 min RLR; *p* < 0.001) decreased; blood loss also decreased (200 cc LLR vs. 150 cc RLR; *p* = 0.046). Conversions to laparotomy almost halved from 9.3% to 4.0% in LLR vs. RLR (*p* = 0.251). No statistically significant differences were observed for LLR vs. RLR in postoperative morbidity (16.5% vs. 26.0%; RR = 1.58; 95% CI 0.90–2.75; *p* = 0.103), major morbidity (4.1% vs. 7.0%; RR = 1.70; 95% CI 0.51–5.62; *p* = 0.538), CCI values (median 0 in both groups; *p* = 0.116) and textbook outcomes (78.4% vs. 79%; RR = 1.01; 95% CI 0.87–1.17; *p* = 0.911). Biliary leaks and PHLF also remained similar in the two groups. Reoperation was never necessary in RLR (vs. 2.1% in LLR). Only one patient of the series (RLR group) deceased because of acute myocardial infarction unrelated to hepatectomy itself. The intra- and postoperative data of LLR and RLR were reported in Table 2.

**Table 2:** Intra- and postoperative data, pathology data in LLR and RLR. Dichotomous variables are expressed as number with percentage. Continuous variables are expressed as mean with standard deviation (SD) or median with interquartile ranges (IQR)

Intraoperative data	LLR (*n* = 97)	RLR (*n* = 100)	*p*-value
Type of index hepatectomy, n (%)			
• Non-anatomic (wedge) resection	62 (64.0)	54 (54.0)	0.636
• Sub-segmentectomy	1 (1.0)	3 (3.0)	
• Segmentectomy	17 (17.5)	23 (23.0)	
• Bisegmentectomy	13 (13.4)	13 (13.0)	
• Left hepatectomy	2 (2.1)	4 (4.0)	
• Right hepatectomy	2 (2.1)	3 (3.0)	
Iwate score, median (IQR)	6 (3–7)	5 (4–7)	0.702
Iwate difficulty level, n (%)			
• Low / Intermediate	27 (27.8) / 32 (33.0)	25 (25.0) / 46 (46.0)	0.247
• Advanced / Expert	30 (30.9) / 8 (8.2)	21 (21.0) / 8 (8.0)	
Multiple resection areas, n (%)	9 (9.3)	32 (32.0)	**< 0.001**
Number of resected areas, median (IQR)	1 (1–1)	1 (1–2)	**< 0.001**
Lymphadenectomy, n (%)	5 (5.2)	9 (9.0)	0.407
Resection of neighbor organs, n (%)	7 (7.2)	9 (9.0)	0.796
Associated local treatment, n (%)	6 (6.2)	13 (13.0)	0.147
Pringle maneuver, n (%)	84 (86.6)	32 (32.0)	**< 0.001**
Total Pringle time (min), median (IQR)	60 (35–85)	35 (19–50)	**< 0.001**
Operative time, median (IQR)	280 (213–364)	263 (194–365)	0.615
Blood loss, median (IQR)	200 (100–300)	150 (100–250)	**0.046**
Conversion, n (%)	9 (9.3)	4 (4.0)	0.251
**Postoperative data**			
Morbidity, n (%) *	16 (16.5)	26 (26.0)	0.103
Major morbidity, n (%) *	4 (4.1)	7 (7.0)	0.538
CCI, mean (SD) and median (IQR)	4.1 (10.5) and 0 (0–0)	6.9 (15.8) and 0 (0.0-8.7)	0.116
Biliary leak, n (%)	2 (2.1)	2 (2.0)	1.000
PHLF, n (%)	1 (1.0)	2 (2.0)	1.000
Reoperation, n (%)	2 (2.1)	0 (0)	0.241
Hospital stay (days), median (IQR)	6 (4–7)	6 (5–8)	0.067
Mortality, n (%)	0 (0)	1 (1.0)	1.000
Textbook outcome, n (%) *	76 (78.4)	79 (79.0)	0.911
**Pathology data**			
R0 resection margin, n (%)	90 (92.8)	91 (91.0)	0.796
Number of tumors resected, mean (SD)	1.3 (0.7)	2.1 (2.0)	**< 0.001**
Largest tumor size (mm)	25.5 (17.0-41.2)	25.0 (16.0–40.0)	0.716
Final pathology, n (%)			
• HCC	32 (33.0)	38 (38.0)	0.579
• CRLM	30 (30.9)	32 (32.0)	
• Gallbladder adenocarcinoma	6 (6.2)	2 (2.0)	
• Intrahepatic cholangiocarcinoma	6 (6.2)	5 (5.0)	
• Non-colorectal liver metastases	13 (13.4)	12 (12.0)	
• Bening tumors	10 (10.3)	11 (11.0)	

CCI, comprehensive complication index; PHLF, post hepatectomy liver failure; HCC, hepatocellular carcinoma; CRLM, colorectal liver metastases. *Overall morbidity: unadjusted risk ratio of 1.58 (95% CI 0.90–2.75). Major morbidity: unadjusted risk ratio of 1.70 (95% CI 0.51–5.62). Textbook outcomes: unadjusted risk ratio of 1.01 (95% CI 0.87–1.17)

## Discussion

This cohort study addressed the risk-adjusted learning curve in MILS of a surgeon trained sequentially in LLR and RLR, a common scenario in liver surgery. The learning curve was based on the CUSUM analysis of multiple relevant continuous outcomes adjusted for patient- and hepatectomy-related confounders.

In the collective cohort of all MILS (LLR and RLR), competency was acquired after 90–111 cases and proficiency after a similar number of cases. Intraoperative outcomes (adjusted operative time and blood losses) improved earlier than postoperative ones (adjusted CCI); this was consistent with the findings of a recent systematic review on learning curves in MILS [15].

Analyzing consecutives LLR and RLR in a unique CUSUM plot enabled a thorough analysis of what happened while transitioning from LLR to RLR in our program. During the implementation of RLR, the adjusted operative times and blood losses continued to improve, while postoperative morbidity remained relatively stable, indicating that no significant deterioration in the surgical performance or postoperative outcomes was observed while introducing the new technique (Fig. 1).

When analyzed separately, a shorter learning curve was observed for RLR following prior LLR experience: the adjusted operative times and blood losses of LLR dropped after 38–45 cases, while for RLR they improved early after 19–29 RLRs. Conversely, the improvement in risk-adjusted postoperative complications was slower and less obvious. This could be related to the low overall complication rates (LLR = 16.5%, RLR = 26%) and to the sample size, which may be still too small to detect a substantial reduction in complications (that are expected to reduce later in the mastery phase of MILS) [15].

The finding of a shorter learning curve for RLR is not a new one and aligned with previous studies [14, 31]. The earlier phase transition observed for RLR may reflect transfer of previous LLR experience, prior exposure to robotic general surgery, cumulative team experience, and potentially platform-related factors. Given the sequential nature of the training in LLR and RLR, the relative contribution of these factors could not be determined. Interestingly, we already showed that in case of concomitant training in LLR and RLR (but adopting an otherwise similar study methodology) the gap between the two learning curves could be smaller [19].

Introducing RLR in our center enabled to expand the use of MILS to 94% of all liver resections within 3 years (supplementary materials, Figure S2), yielding a rate of textbook outcomes of 79%, equal to LLR. The latter finding is particularly relevant as it is consistent with contemporary large single and multicentric series including over 600 RLRs [5, 32]. These results further confirmed the successful introduction of RLR while maintaining high standards of care.

From a methodological standpoint Kuemmerli et al. stressed the need for standardized reporting of learning curve in MILS. Out of 102 learning curves examined, only 60% were obtained from statistical calculations (i.e., CUSUM in 35%), 41% targeted multiple endpoints or variables, and very few used risk-adjustments (8%). The authors proposed the 3-phase learning curve model endorsed in the present study [15].

We adopted an easily reproducible, risk-adjusted learning curve methodology, based on three clinically relevant outcomes. Compared with more standardized and less demanding operations (i.e., hernia repair), risk-adjustments are essentials in MILS, as patient and disease factors influence the clinical outcomes alongside the technical complexity of hepatectomy [33–35]. Moreover, although a single primary outcome is ideal for clarity, the use of multiple risk-adjusted ones allowed complementary assessment of different dimensions of the surgical performance [36–38].

Like in our study, Halls and colleagues adjusted for confounders through a regression analysis and analyzed the risk-adjusted learning curve of LLR in pioneers and early adopters with the CUSUM method. They estimated a learning phase of 46 LLRs for early adopters; however, the study did not include any RLR [12]. The meta-regression analysis by Chua et al. suggested thresholds of 50 (range 25–58) LLRs and 25 (range 16–50) RLRs to surpass the first phase of the CUSUM-based learning curves. However, only 7 studies investigated the learning curve of RLR, and none adjusted for confounders [14]. Krenzien et al. used the CUSUM analysis of the operative time normalized for the Iwate scores to measure the risk-adjusted learning curve in a center transitioning from LLR to RLR. The authors identified similar learning phases in LLR (*n* = 117) and RLR (*n* = 93). Beside the larger sample (LLR = 514, RLR = 132), the adjustments were based solely on the Iwate scores and included high numbers of major hepatectomies especially for RLR (LLR = 25.6% and RLR = 47.3%). This possibly led to different results (much longer learning curves) than in our study [20].

Regular access to robotics is another relevant and usually underestimated confounding factor. Venezia et al. recently analyzed the learning curve of 650 RLRs from 10 French referral centers for liver surgery, all moving from LLR to RLR across a 14-year timespan. The authors showed that improvements in conversions and blood losses were achieved faster in centers with weekly access to RLR. Notably, the CUSUM analysis was not risk-adjusted and was weighted only by center RLR volume. The latter was heterogenous and overall low (around 11 RLR/year/center, with just two centers having performed > 100 RLRs overall), warranting careful interpretation of the results [38].

This study carried some limitations. First, despite a robust and updated methodology, residual selection bias could not be completely excluded due to the retrospective design of the study, moreover, as LLR and RLR were performed during different periods, cumulative surgical and team experience may have also contributed to the observed differences. Risk-adjusted CUSUM-based learning curves are relatively easy-to-understand and commonly accepted; however, they rely on the average surgical outcome of the series examined and may be prone to subjective interpretation [39]. Specifically, risk-adjusted models for LLR and RLR included 11 covariates in approximately 100 observations and should therefore be interpreted cautiously, particularly for blood loss and CCI, which showed lower model fit. The phase-boundaries identified in the study should be interpreted as approximate transition points in risk-adjusted surgical performance rather than exact statistical thresholds of surgical competence. Second, in our series there were few major hepatectomies (i.e., left or right hepatectomy) and cases with biliary or vascular reconstruction were absent. Studies involving populations with a larger amount these types of resections could produce different results. Third, the duration of the learning curve correlates with the size of the population examined: studies with similar methodology but different size could also lead to different results.

## Conclusions

A shorter learning curve was observed for RLR following prior experience in LLR, with risk-adjusted improvements occurring earlier in operative time and blood loss than in postoperative morbidity for both approaches. The careful stepwise introduction of RLR did not deteriorate the surgical performance within the transition from LLR to RLR. Patient and disease complexity influence the outcomes of MILS alongside technical complexity and must be considered for reliable risk-adjusted learning curve assessments.

## Supplementary Information

Below is the link to the electronic supplementary material.

Supplementary Material 1

## Data Availability

No datasets were generated or analysed during the current study.
