# Supplementary material for: Transition from laparoscopic to robotic hepatectomy within a minimally invasive liver surgery program: risk-adjusted learning curve assessment by multiple continuous outcomes
Source: J Robot Surg. 2026 Sep 27;20(1):1028. doi: 10.1007/s11701-026-04004-1 (PMC13616854; doi:10.1007/s11701-026-04004-1)
Supplement: Supplementary file 1 [file 11701_2026_4004_MOESM1_ESM.docx]

**Supplementary Materials**

**Figure S1.** Flowchart and design of the study.

**Figure S2.** Stacked area graph showing the transition from LLR to RLR and the decrease of OLR over time in our center.

Legend: Y axis, proportions of liver resection; X axis, years within the study period.

Colors: grey = OLR (open liver resection); blue = LLR (laparoscopic liver resection); red = RLR (robotic liver resection).

**Table S1.** Model fit and residual diagnostics.

| Cohort | n | Outcome | R^2^ | Adjusted R^2^ | Residual SD | Shapiro-Wilk p-value |
| --- | --- | --- | --- | --- | --- | --- |
| MILS | 197 | Operative time | 0.522 | 0.493 | 0.139 | 0.199 |
|  |  | Blood loss | 0.171 | 0.122 | 0.769 | <0.001 |
|  |  | CCI | 0.124 | 0.072 | 0.532 | <0.001 |
| LLR | 97 | Operative time | 0.501 | 0.436 | 0.139 | 0.468 |
|  |  | Blood loss | 0.224 | 0.124 | 0.709 | <0.001 |
|  |  | CCI | 0.137 | 0.025 | 0.483 | <0.001 |
| RLR | 100 | Operative time | 0.616 | 0.568 | 0.127 | 0.137 |
|  |  | Blood loss | 0.224 | 0.127 | 0.773 | <0.001 |
|  |  | CCI | 0.201 | 0.101 | 0.544 | <0.001 |

**Table S2.** Three-phase risk-adjusted learning curve of MILS based on our experience: a) Risk-adjusted learning curve phases visually identified; b) Sensitivity analysis (local segmented regression).

| **Group** | **n** | **Variable/outcome** | a) **Risk-adjusted learning curve phases visually identified** | | |
| --- | --- | --- | --- | --- | --- |
|  |  |  | **Competency, cases** | **Proficiency, cases** | **Mastery, cases** |
| **All MILS** | 197 | Adjusted operative time | 0-90 | 91-182 | From case 183 on |
|  |  | Adjusted blood loss | 0-111 | 112-165 | From case 166 on |
|  |  | Adjusted CCI | 0-93 | 94-165 | From case 166 on |
| **LLR** | 97 | Adjusted operative time | 0-38 | 39-80 | From case 81 on |
|  |  | Adjusted blood loss | 0-45 | 46-91 | From case 92 on |
|  |  | Adjusted CCI | 0-40 | 41-94 | From case 95 on |
| **RLR** | 100 | Adjusted operative time | 0-19 | 20-63 | From case 64 on |
|  |  | Adjusted blood loss | 0-29 | 30-70 | From case 71 on |
|  |  | Adjusted CCI | 0-21 | 22-92 | From case 93 on |
| **Group** |  | **Variable/outcome** | b) **Sensitivity analysis (local segmented regression)** | | |
| **All MILS** | 197 | Adjusted operative time | 0-87 | 88-181 | From cases 182 on |
|  |  | Adjusted blood loss | 0-112 | 113-167 | From case 168 on |
|  |  | Adjusted CCI | 0-94 | 95-166 | From case 167 on |
| **LLR** | 97 | Adjusted operative time | 0-35 | 36-77 | From case 78 on |
|  |  | Adjusted blood loss | 0-47 | 48-91 | From case 92 on |
|  |  | Adjusted CCI | 0-39 | 40-91 | From case 92 on |
| **RLR** | 100 | Adjusted operative time | 0-17 | 18-60 | From case 61 on |
|  |  | Adjusted blood loss | 0-27 | 28-60 | From case 61 on |
|  |  | Adjusted CCI | 0-18 | 19-92 | From case 93 on |
